# Fireside Science: A Series of Popular and Scientific Essays upon Subjects Connected with Every-day Life

**Published:** 1872-03

**Authors:** 


### Books Reviewed.

*Fireside Science: a series of popular and Scientific Essays upon subjects connected with every-day Life.* By James R. Nichols, A.M., M.D. New York: Hurd & Houghton, 1872. The Riverside Press, Cambridge.

This is a handsome collection of popular Essays upon scientific subjects of interest to the popular mind. The opening essay is upon the subject of the Origin and Nature of Springs. It gives the geological formations observed where springs abound, the different varieties of springs, and the locality of the most note-worthy. The chemistry of a Cigar, and of a pint of Kerosene are, each in their turn, the subject of an essay, and will prove of interest to the consumers of those articles. The article on the Lost Arts, though perhaps not so extended and searching as the lecture of Wendell Philips upon the same subject, is more adapted to the younger minds of the "Fireside Circle."

The Chemistry of the Human Body, and Infectious Germs, are articles which will, no doubt, be of more immediate interest to the medical man; although not as extended and minute as a purely scientiffc essay would be.

Dr. Nichols has, in a few well chosen words, said a great many things which will be of interest to both old and young in the "Family Circle."

